# Development of an automated biomaterial platform to study mosquito feeding behavior

**DOI:** 10.3389/fbioe.2023.1103748

**Published:** 2023-02-09

**Authors:** Kevin D. Janson, Brendan H. Carter, Samuel B. Jameson, Jane E. de Verges, Erika S. Dalliance, Madison K. Royse, Paul Kim, Dawn M. Wesson, Omid Veiseh

**Affiliations:** ^1^ Department of Bioengineering, Rice University, Houston, TX, United States; ^2^ Department of Tropical Medicine, Tulane University, New Orleans, LA, United States

**Keywords:** machine learning, object detection, biofabrication, 3D printing, mosquito-borne diseases, mosquito repellent

## Abstract

Mosquitoes carry a number of deadly pathogens that are transmitted while feeding on blood through the skin, and studying mosquito feeding behavior could elucidate countermeasures to mitigate biting. Although this type of research has existed for decades, there has yet to be a compelling example of a controlled environment to test the impact of multiple variables on mosquito feeding behavior. In this study, we leveraged uniformly bioprinted vascularized skin mimics to create a mosquito feeding platform with independently tunable feeding sites. Our platform allows us to observe mosquito feeding behavior and collect video data for 30–45 min. We maximized throughput by developing a highly accurate computer vision model (mean average precision: 92.5%) that automatically processes videos and increases measurement objectivity. This model enables assessment of critical factors such as feeding and activity around feeding sites, and we used it to evaluate the repellent effect of DEET and oil of lemon eucalyptus-based repellents. We validated that both repellents effectively repel mosquitoes in laboratory settings (0% feeding in experimental groups, 13.8% feeding in control group, *p* < 0.0001), suggesting our platform’s use as a repellent screening assay in the future. The platform is scalable, compact, and reduces dependence on vertebrate hosts in mosquito research.

##  Introduction

Mosquito-borne pathogens that cause diseases such as dengue, malaria, Zika, and yellow fever have earned mosquitoes the title of “the world’s deadliest animal” to humans because of the number of people who die each year from mosquito-borne infections (Kamerow, 2014). These diseases disproportionately impact Asia, Africa, and South America, and incidence is linked with poverty (Gallup and Sachs, 2001; Bhatt et al., 2013). Most female mosquitoes require the proteins in blood to develop eggs (Woke et al., 1956) with rare exceptions (Engelmann, 1970; Ariani et al., 2015), and infected female mosquitoes transmit pathogens while feeding on human blood. The burden posed by mosquito-borne pathogens presents the need for studies that investigate mosquito feeding behavior in a controlled environment, with a goal of better understanding the feeding process and ultimately finding ways to decrease pathogen transmission rates (Frances and Debboun, 2022). There is therefore a need to develop a high-throughput assay compatible with multiple mosquito species to screen the effectiveness of different mosquito repellents. Since the creation of DEET in 1946, a small handful of comparably effective repellents have been developed, although DEET is still considered to be the gold standard for mosquito repellents (Katz et al., 2008). Although new repellent candidates are actively being investigated (Zhu et al., 2018), novel repellent development has been slow because of the costs associated with regulatory approval, challenges with entering an established market, and limitations in testing throughput for new repellent compounds. To decrease the dependence on humans and animals during repellent discovery and address the issue of testing throughput, we investigate the utility of biocompatible hydrogels as skin mimics to observe mosquito feeding.

In recent years, advances in 3D bioprinting have enabled high-resolution patterning of vascular structures in biocompatible materials (Grigoryan et al., 2019). Additionally, these materials often support perfusion of blood to further mimic biological tissue. These advances enable researchers to substitute native or explanted tissues with synthetic alternatives for certain applications, thereby reducing cost and limiting ethical concerns. Avoiding animal subjects is particularly useful for mosquito research, which has historically used live animals or humans as a food source (Ribeiro, 2000; Ross et al., 2019) during studies that investigate mosquito repellents to decrease the frequency of mosquito bites (Carroll and Loye, 2006; Haris et al., 2022; Hazarika et al., 2022). High-throughput enabled discovery of new repellents would greatly alleviate testing bottlenecks (Klun et al., 2005). Subsequent regulatory approval and product promotion could then bring new repellents to market to alleviate the nuisance of mosquito bites, mitigate the spread of certain pathogens, and decrease patient morbidity and mortality.

Although several assays have been proposed to screen mosquito attractants (Kim et al., 2021a; 2021b) or repellents (Grieco et al., 2005; Tisgratog et al., 2016; Kajla et al., 2019; Chauhan et al., 2021), creating a controlled environment to simultaneously test the impact of multiple variables such as temperature and blood type on mosquito feeding behavior remains challenging. Several recently published experimental platforms effectively quantify repellent effectiveness, but often rely on human volunteers or use spatially inefficient designs, thereby limiting their scalability (Goodyer et al., 2020; Farooq et al., 2022). Making a hydrogel model that adequately represents skin requires optimizing hydrogel composition, vascular architecture, and choice of perfused fluid, as different mosquito species display preferences for certain host species (Ribeiro, 2000). To expand applicability, a mosquito-feeding assay would ideally be compatible with several species because most mosquito-borne pathogens are specific to certain mosquito species (Verhulst et al., 2011).

A challenge of developing a high-throughput screening method of any kind is processing and analyzing large amounts of data. Fortunately, computer vision has recently emerged as a powerful tool for tracking objects through space in real time (Brunetti et al., 2018; Chandan et al., 2018; Feng et al., 2019; Zhou et al., 2020; Bjerge et al., 2022) and classifying objects into predetermined categories (Tan, 2004; Issac et al., 2017). These capabilities indicate the utility of computer vision for automatically identifying objects like mosquitoes within video frames. Some groups have already successfully used computer vision to automatically track mosquito behavior and observe feeding patterns (Hol et al., 2020). The potential of computer vision for processing videos increases when combined with machine learning algorithms to discern feeding and non-feeding mosquitoes from background video footage.

Here we create a platform for studying mosquito feeding behavior by integrating several technologies into a single system. We used carefully designed single-use hydrogels perfused with blood to elicit mosquito feedings, collected data in the form of video recordings, and analyzed that data using computer vision techniques. Our platform is compatible with different perfused fluids, which is useful for examining different sources of blood as well as artificial food sources for mosquitoes (Gonzales and Hansen, 2016). These perfused fluids can be adjusted along with different mosquito species, therefore expanding the platform’s experimental applicability. Finally, we used our developed mosquito feeding platform to evaluate the effectiveness of two repellents, suggesting its potential as a high-throughput screening platform with further scale-up.

##  Materials and methods

###  Hydrogel design and fabrication

Hydrogels containing a vascular network were designed using Blender, an open-source 3D CAD software (Blender Foundation, Amsterdam, Netherlands). Designs were exported in STL format, and photomasks were generated using Creation Workshop software (https://makershop.co/envision-labs/). These photomasks were modified using a custom MATLAB script that adjusted the grayscale value of photomasks in select regions to 60% intensity. The resulting custom photomasks were used to fabricate hydrogels *via* 3D printing.

Hydrogels were fabricated as previously described (Grigoryan et al., 2019) with some modifications. Pre-hydrogel solution was prepared using 3.25% wt/vol 3.4 kDa poly(ethylene glycol) diacrylate (PEGDA), 10% wt/vol gelatin methacrylate (GelMA), 10% wt/vol glycerol, 17 mM Lithium phenyl-2,4,6-trimethylbenzoylphosphinate (LAP), and 3.25 mM tartrazine (Sigma-Aldrich, St. Louis, MO, United States). This solution was 3D printed *via* digital light processing (DLP) on an Lumen X bioprinter (Cellink, Boston, MA, United States). The layer height for printing was fixed at 50 μ m and the bioink was exposed to light for 12.0 s per layer with approximately 21 mW/cm^2^ in intensity. Three hydrogels were printed simultaneously for a total print time of 23 min. Following printing, hydrogels were allowed to remain in PBS for a minimum of 2 days before experiments were performed. PBS was replaced several times as excess tartrazine leached out of the hydrogels until the PBS solution was completely clear. To make a model system that would enable mosquito feeding, hydrogels with a simple vascular architecture were used to mimic a skin sample. All of the hydrogels described here were used in one experiment and were discarded after use.

###  Defibrinated blood perfusion

Research-grade defibrinated animal blood was purchased from HemoStat Laboratories (Dixon, CA, United States). All feeding experiments used either defibrinated chicken, sheep, or cow blood. Blood was loaded into a 30 mL syringe and was placed in a syringe pump (New Era Pump Systems, Inc., Farmingdale, NY, United States). Blood was flowed at a rate of 100 μ L/min through Tygon^®^ tubing (Cole-Parmer, Vernon Hills, IL, United States) for the duration of all mosquito feeding experiments. Tight fluidic connections were achieved *via* blunt syringe tips (Nordson Corporation, Westlake, OH, United States). Blunt 90-degree needle tips were modified by removing the metal tip from the luer lock connector using pliers, and tubing was connected directly to the detached metal tip. These tips and the hydrogels were held in place with 3D printed perfusion chambers printed using poly(lactic acid) (PLA) filament (Ultimachine, South Pittsburg, TN, United States) on a Prusa i3 MK3S+ printer (Prusa Research, Prague, Czech Republic). Perfusion chambers were designed using a pre-defined protocol (Kinstlinger et al., 2021) with subsequent modifications using Fusion 360 software (Autodesk, San Rafael, CA, United States). Tubing was used to connect multiple hydrogels in series such that a single syringe pump could supply blood flow to up to six hydrogels.

###  Platform assembly

Printed circuit boards (PCBs) (Conclusive Engineering, Katowice, Poland) were designed with six evenly spaced sites for attachable hydrogels arranged in a 3 × 2 grid. Each site contained a resistive heater, LEDs, and a thermistor. The settings for the heating elements and LEDs were controlled using custom software. Individual heating elements and LEDs were independently controlled according to the requirements of particular experiments. The platform was positioned vertically and held in place with a combination of custom 3D printed parts, T-slotted framing rails, and nuts and bolts. The 3D printed perfusion chambers securing needle tips and hydrogels were bolted to the PCBs. These chambers were printed using white filament to provide contrast against the black PCBs in an effort to attract mosquitoes. Machined parts were purchased from McMaster-Carr (Atlanta, GA, United States). Raspberry Pi 4 Model B computers were outfitted with PoE+ HAT attachments to support power delivery and data communication among Raspberry Pis *via* ethernet cables. Both the Raspberry Pi computers and PoE+ HATs were purchased from Adafruit (New York, NY, United States). A PoE+ network switch (Netgear, San Jose, CA, United States) supplied power to all Raspberry Pi computers through the ethernet cables. Raspberry Pi camera boards and lenses (Arducam, Nanjing, China) were fixed in place with adjustable 3D printed parts. Each Raspberry Pi camera was directed at a single hydrogel housed in a perfusion chamber. Raspberry Pis recorded video directly to their respective hard drives using custom Python scripts.

###  Mosquito rearing

Feeding experiments were performed using lab raised *Aedes aegypti* (*Ae. aegypti*) Rockefeller strain mosquitoes. The mosquitoes were raised in a controlled insectary at 25°C–27°C, 75%–80% relative humidity and a photoperiod of 16:8 (light:dark) hours. Dried egg papers less than 3 months old were hatched in deoxygenated water and transferred to polypropylene Nalgene pans at a density of 200 larvae/2 L water and fed on algae tablets (Hikari, Kyorin Food Ltd. Japan). Pupae were transferred to beakers and placed in cages to emerge; approximately 500 adult males and females were kept in 30 × 30 × 30 mesh cages (BugDorm, MegaView Science Co., Ltd. Taiwan) and allowed to mate freely. Adults were provided 10% wt/vol sucrose solution delivered by cotton wick. Sucrose solution in select cages was removed overnight prior to feeding experiments.

###  Feeding experiments

Hydrogels were loaded into 3D printed perfusion chambers and tight fluidic connections were created to support perfusion (see above). These perfusion chambers were bolted to a PCB, which was held in place vertically. After flowing blood through all hydrogels to ensure their structural integrity, a solid glass cage was fitted over the PCB and 20–30 female mosquitoes were introduced into the cage through a hole. Mosquitoes were selected by mechanical aspiration of the subset of the colony attracted to a human hand held close to a mesh wall of the colony cage. Several male mosquitoes were included with aspirated females to encourage feeding. The cage’s edges did not allow mosquitoes to escape, and the hole was plugged with a cotton ball after mosquitoes were fully released into the cage. A Raspberry Pi camera was aimed at each hydrogel so that mosquito behavior and feeding could be recorded. Feeding experiments were stopped after 30–45 min of activity and mosquitoes were subsequently isolated for manual quantification of feeding and egg counts.

###  Meal choice experiments

A total of 3 mosquito feeding cages were prepared the same way as for feeding experiments, with minor changes. Hydrogels in each of the three cages were perfused with either defibrinated blood, red India ink, or PBS. The hydrogels in a given cage all received the same liquid, and each of the three liquids was heated to 37°C before entering the cage. Approximately 20–30 *Ae. aegypti* mosquitoes were introduced as normal and were observed for feeding behavior.

###  Repellent screening experiments

Similar to meal choice experiments, 3 feeding cages were prepared with distinct experimental conditions. One of the cages contained 6 hydrogels coated with 10 mg/mm^2^ of 25% DEET (N,N-diethyl-meta-toluamide), another contained 6 hydrogels coated with 10 mg/mm^2^ of a 30% concentration of a plant-based repellent derived from the oil of lemon-eucalyptus plants (OLE), and the last cage contained 6 uncoated hydrogels and served as a control. All hydrogels were perfused with blood that was heated to 37 °C before entering the cage. 20–30 female *Ae. aegypti* mosquitoes were introduced into each cage and activity was observed for 30–45 min. The mosquitoes introduced to the control, DEET, and OLE groups were selected from a population of approximately 500 mosquitoes hatched on the same day based on their attraction to a human hand held near a mesh wall of the colony cage. Repellent experiments were repeated for five total replicates to reduce noise due to sample differences in feeding activity. Each experimental replicate used a different population of mosquitoes to minimize batch effects.

###  Machine learning dataset construction

Following the platform assembly and blood perfusion methods described above, videos of mosquito feedings were collected. During the early stages of model development, approximately 50 still images were extracted from these videos. These images were pre-processed by downsizing to 416 × 416 pixels to reduce computation time and by disregarding images that do not contain any mosquitoes. Training data were supplemented with augmented images that contained several transformations (see Supplementary Methods) to improve model performance. Images were manually labeled with bounding boxes for mosquitoes, abdomens of feeding or engorged mosquitoes, and abdomens of non-feeding mosquitoes (see Supplementary Methods). A machine learning model was trained and deployed *via* a custom algorithm developed by Roboflow, Inc. (San Francisco, CA, United States, see Supplementary Methods), and performance was evaluated based on precision, mean average precision (mAP), and recall. New images were labeled and added to the model as experiments progressed. Initial training data was heavily skewed toward representing non-feeding mosquitoes, as the number of frames containing feeding or engorged mosquitoes was drastically smaller. To compensate for this issue, the model was deployed on unlabeled videos to identify images containing feeding or engorged mosquitoes. These images were automatically uploaded to the dataset for future incorporation if they were less than 97% similar to previously analyzed frames. Our final dataset consisted of 3,292 images, which were segmented into training, validation, and testing datasets in a ratio of 7:2:1.

###  Derivation of mosquito activity metric

Machine learning model outputs were reduced to a single number per label type (i.e. mosquito, non-feeding, and feeding) for each video to quantify activity. Raw counts of each label’s abundance were tabulated for each frame, creating a function of label abundance over time. These counts were then summed to obtain the total number of labels throughout a video. This metric can be represented by the following expression:

$$Activity=\overset{n}{\underset{i=1}{\sum }}D\left(t\right)$$

Where *n* represents the number of frames in a video and *D(t)* represents the number of model detections at time *t* for a given class. Because mosquito presence and incidence of biting are the most relevant indicators of repellent effectiveness, we chose to only analyze mosquito and feeding labels with our metric. In some cases (noted in Results) we limited our video data to a region of interest that included hydrogels and their immediate surroundings to ignore mosquitoes within the field of view but are far away from hydrogels. To decrease computation time and resources, videos were often uniformly downsampled by a factor of 16 (i.e., every 16th frame was kept). Our current computational setup allows us to use our model to interpret videos at a rate of 1.67 frames per second, and that analysis rate could be amplified by running several analyses in parallel on different machines.

###  Hydrogel imaging

Hydrogels were fabricated as described above. To visualize the vascular channel and verify its patency and distance from the hydrogel surface, a solution containing a 1:200 dilution of red fluorescent beads (Magsphere, Pasadena, CA, United States), Irgacure 2959 (Ciba), and 20% wt/vol 6 kDa PEGDA with PBS as a diluent was injected into the channel and crosslinked under UV light. The hydrogel was then inserted into a 3D printed chamber and carefully sliced with a razor blade. The 3D printed chamber featured slots for the razor blade for the sake of consistency. The hydrogel cross sections were imaged on a Ti-E inverted microscope (Nikon, Melville, NY, United States) and a Zyla 4.2 sCMOS camera (Andor, Belfast, United Kingdom). The resulting images were linearly adjusted for brightness and contrast to improve visualization.

Based on the results of pilot experiments, hydrogels were softened in precisely patterned regions directly above the vascular channels to ease mosquito feeding. To visualize the differences between these patterned regions (termed “compliant regions” for their increased compliance) and unaltered regions on hydrogel surfaces, pre-hydrogel solution was supplemented with FITC conjugated to 150 kDa dextran at a concentration of 1 mg/mL. Hydrogels were printed in sets of three, each of which contained softened compliant regions at regular intervals on hydrogel surfaces. For each set, one of these hydrogels was sliced and imaged (see above) immediately after printing, and the remaining two were stored in PBS and were imaged at 2 and 5 days post-printing, respectively. Hydrogels were imaged using the same exposure settings for all three timepoints, and brightness was uniformly adjusted to assist visualization. Differences of hydrogel fluorescence intensity between compliant and adjacent control regions of hydrogels were compared at 0, 2, and 5 days post-printing. Quantification of fluorescence intensity was performed by summing the pixel intensity within a consistently sized rectangular region over compliant regions and control regions of the hydrogel. Every compliant and control region was measured this way, and regions within the same hydrogel were treated as technical replicates.

###  Statistical analysis

Statistical analyses of all data were performed using R software and GraphPad Prism 9.4. Data for the diffusion assay were collected for N = 6 hydrogels on days 0 and 2 post-printing, and for N = 5 hydrogels on day 5 post-printing. It was determined that fluorescence readings were not normally distributed after day 0 (*p* < 0.01, Shapiro-Wilk test), so measurements were compared using a non-parametric Mann-Whitney test for each day. The data are shown in Figure 3 as mean ± S.D. For experiments consisting of multiple feeding cages with isolated conditions (i.e. meal choice experiments and repellent screening experiments), a chi-square test was used to compare feeding preferences of multiple mosquito populations. Comparisons of egg counts between mosquitoes feeding on hydrogels (N = 85) vs the laboratory standard as a control (N = 45) were performed using a two-tailed unpaired *t*-test (*p* > 0.05) after confirming that the data for both groups was sufficiently normally distributed (*p* > 0.05, Shapiro-Wilk test).

##  Results

###  Platform development

**FIGURE 1:**
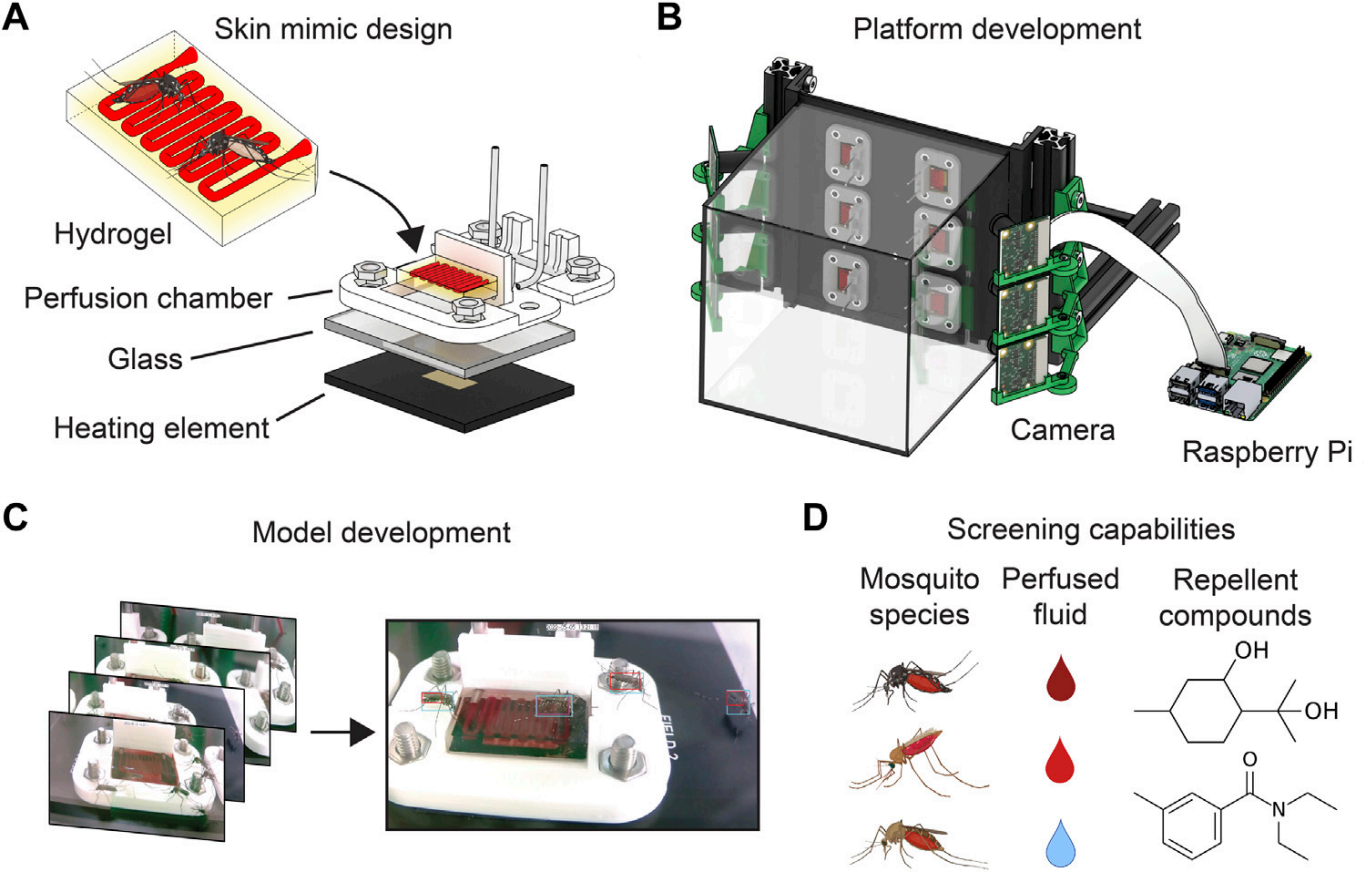
Development of a repellent screening platform. **(A)** Hydrogels are set within a perfusion chamber (white) connected to 90° metal blunt needle tips to support blood flow (red). The chamber is positioned over glass which acts as a barrier between the hydrogel and the heating element on a PCB. **(B)** Up to six chambers are bolted to the PCB (black), which is fitted with a glass cage. PCBs are positioned vertically and are held in place by aluminum T-slotted framing rails. Six raspberry Pi computers (one shown) are each connected to a camera aimed at each hydrogel. **(C)** Raspberry pi video data is used to develop a machine learning model that identifies mosquitoes and their feeding behavior. Photographs have been white balanced for visualization purposes. **(D)** Our feeding platform is compatible with different mosquito types and has been verified to work with *Aedes, Anopheles,* and *Culex* mosquitoes. The platform is also compatible with different perfused fluids and can be used to evaluate the effectiveness of different repellents. After developing a satisfactory machine learning model, we use our system to screen different repellents for their ability to prevent mosquito feeding.

The hydrogels that served as a mosquito food source consisted of a single channel that sweeps back and forth several times in a serpentine pattern (Figure 1A). The switchbacks of the serpentine were spaced far enough apart so that rupturing during perfusion was unlikely, but close enough to maximize the available feeding area for mosquitoes. After several iterations of testing, it was determined that vessels 400 μm from the feeding surface would consistently print and support perfusion while still enabling mosquitoes to easily reach the artificial blood vessel during feeding.

To gather a rich dataset of mosquito feeding behavior, we sought to record videos of mosquitoes feeding on our hydrogels. After several rounds of optimization (see Supplementary Methods), we adopted a system that allowed us to record videos to a series of Raspberry Pi computers that allow recording at 1920 × 1080 resolution at 30 frames per second (fps) while occupying approximately 2.5 L worth of space (Figure 1B). Recording video data of mosquito feeding experiments enabled us to develop a machine learning model and perform experiments with multiple mosquitoes, perfused liquids, and surface repellents (Figure 1C, D).

It has been well documented that mosquitoes rely on a variety of cues to find hosts, one of which is heat (Potter, 2014; Liu and Vosshall, 2019). We therefore hypothesized that heating hydrogels during experiments could improve mosquito attraction to our skin mimics. Additionally, illuminating hydrogels might enhance photography and data collection, although colored light may also affect mosquito attraction (Gjullin, 1947; Van Breugel et al., 2015; Liu and Vosshall, 2019; Alonso San Alberto et al., 2022). To incorporate heating and lighting capabilities into our platform, we designed a custom printed circuit board (PCB) with heating elements and LED lights at six fixed positions. Our preliminary experiments revealed that mosquitoes feeding on a horizontal surface will orient themselves at a variety of body angles relative to a stationary camera, but mosquitoes feeding on a vertical surface tend to orient themselves so that their abdomens are pointing down. To support a vertical feeding surface, our PCB features through holes to enable bolting hydrogel perfusion chambers to it. This capability improved consistency of hydrogel placement and firmly anchored them to the PCB, allowing us to position the entire PCB vertically. Video data suggested that mosquitoes were more likely to orient themselves the same way on this vertical surface, so this angle was maintained for future experiments.

Next, we leveraged our PCB’s heating capabilities to quantify the impacts of heat on mosquito attraction. Prior to performing experiments, we empirically determined that PCBs were able to heat hydrogels to a maximum temperature of 34 °C, which is approximately equal to skin temperature (Metzmacher et al., 2018). We then designed an experiment that offered mosquitoes an equal choice of three unheated hydrogels and three hydrogels heated to 34 °C to see whether heating increased attraction. After introducing groups of laboratory-reared *Ae. aegypti* mosquitoes, we observed that the mosquitoes appeared distracted by the perfusion chambers and nuts and bolts securing the chambers to the PCB and exhibited host-seeking behavior on these surfaces as well. We hypothesize that heat transfer inefficiencies heated up hardware surrounding the hydrogels, resulting in unexpected mosquito attraction to these surfaces. Surprisingly, repeating this experiment with *Anopheles quadrimaculatus* (*An. quadrimaculatus*) mosquitoes yielded less distracted behavior and revealed that heated hydrogels are required to elicit feeding for this species (Supplementary Figure S1). We therefore concluded that our PCB’s heating successfully increased mosquito attraction in some species but was experimentally detrimental for others.

###  Computer vision quantification

**FIGURE 2:**
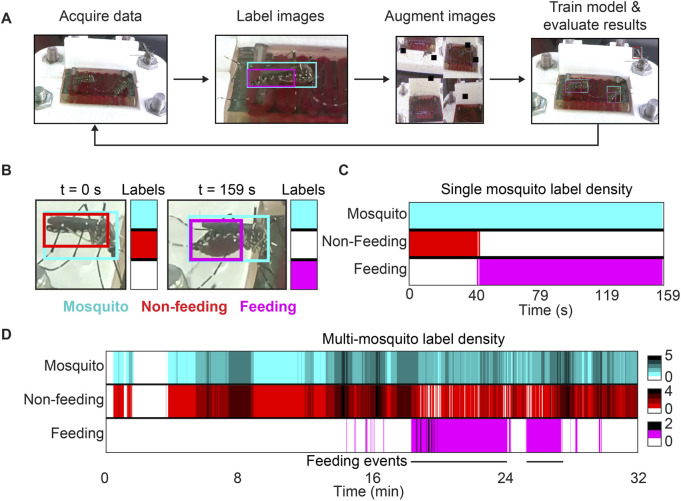
Training workflow and results for our machine learning model. **(A)** Overview of the pipeline used to develop a computer vision model. Acquired video data was segmented into still frames in which mosquitoes, abdomens of feeding or engorged mosquitoes, and abdomens of non-feeding mosquitoes were manually labeled. Training data was supplemented using augmented images (see supplementary methods). Performance statistics were evaluated after training and the model was iteratively improved. **(B)** Within a video containing multiple feeding or engorged mosquitoes, a single one is shown at the beginning and end of feeding. The model predicts that the mosquito is not feeding when its abdomen is small and predicts that it is feeding when its abdomen is large and red. Model labels at any given time can be represented by a 3 × 1 colorized vector, shown next to the two images in this panel. **(C)** Model predictions for the mosquito shown in **(B)** were recorded for every frame during a feeding event. For each timepoint, the colorized vectors described in **(B)** were displayed as a continuum. The mosquito is initially identified as non-feeding, but that classification switches to “feeding” as the abdomen’s appearance changes. **(D)** Model outputs for the same video shown in **(B–C)**, but without constraining predictions to a single feeding event. Density gradients have been added to represent the abundance of model predictions at any given time. For this video, two distinct feeding events can be visualized by two solid and consistent magenta bands representing feeding labels.

After developing a feeding platform and establishing a consistent experimental procedure, a total of over 180 recordings of mosquito feeding experiments were collected over a period of 6 months. These videos were used to train a machine learning object detection model to identify mosquitoes (Figure 2A). Some videos exhibited slightly different camera angles and lighting, which is beneficial because it does not overfit to a particular experimental setup, expanding the applicability of the model to a variety of laboratory settings. This machine learning model was developed using *Ae. aegypti* mosquitoes during experiments that did not use PCB heating.

Our object detection model was assessed by applying it to video files and analyzing the returned predictions. We first trained our model to identify mosquitoes within the camera’s field of view. Next, we further characterized the behavior of visible mosquitoes by training the model to identify two additional objects: feeding/engorged mosquito abdomens and non-feeding mosquito abdomens. When assessed in aggregate, the model’s mean average precision (mAP) of 92.5% implies that the model performs well across all three classes (see Supplementary Methods). The model’s relatively high recall of 89.2% indicates that it has a low false negative rate, and its 92.1% overall precision indicates that it has a low false positive rate as well. In the case of precision, more meaning can be extracted when examining each class individually.

The developed model can identify mosquitoes with 98% precision, meaning that 98% of all mosquito detections made by the model are accurate. The effectiveness in distinguishing feeding or engorged from non-feeding mosquitoes was assessed using the detection classes for feeding and non-feeding mosquito abdomens (see methods). Using this approach, the model was able to correctly identify feeding or engorged mosquitoes with 94% precision and non-feeding mosquitoes with 90% precision. It should be noted that these classes are treated independently, so it is possible for a single mosquito to be labeled as both feeding and non-feeding simultaneously. However, if these classes are combined into a single “abdomen” label regardless of feeding status, the model identifies abdomens correctly with 96% precision. Although the model makes some mistakes, it often performs well in challenging situations such as when mosquitoes are partially obscured or out of focus.

After developing a model that accurately identifies three objects of interest, we sought to use this model to automatically parse mosquito feeding videos and extract meaning from them. The performance statistics we obtained from our model were impressive, but it was unclear whether the model’s inaccuracies would render it useless for analyzing videos. Starting with small test cases, we first evaluated the model’s performance on trimmed videos that were cropped to only show a single feeding mosquito. Overall, mosquitoes analyzed using this method were identified by the model as non-feeding while their abdomens remained slim and feeding when their abdomens became swollen and red (Figure 2B). Visualizing model predictions for an entire video revealed the existence of a transition period, during which time the abdomen was classified as both feeding and non-feeding simultaneously (Figure 2C). This transition period roughly corresponds to the time during which a human observer would have difficulty telling whether a mosquito has begun to feed or not. Finally, once the abdomen undergoes substantial change from its original state, the model consistently identifies the abdomen as that of a feeding mosquito.

While we were pleased with our model’s predictions for individual mosquitoes over the course of feedings, limiting analysis to a particular ROI requires manual intervention and prior knowledge of where feeding mosquitoes are in a video. Although this intervention is minimal, it still limits the throughput and applicability of our technology. To further improve automation, we applied our model to an entire unmodified video to return raw counts of each label per frame (Figure 2D). We displayed the data in a similar way as with individual mosquitoes, but added color gradients to show label density throughout time. This new visual representation reveals the density of mosquitoes and feeding events for any given frame and elucidated trends in mosquito behavior for feeding experiments. It is also possible to visualize the time and duration of specific feeding events if they are temporally separated. This visualization tool provides informative qualitative and quantitative data about collected videos and obviates the need for researchers to manually watch videos to make similar observations.

###  Hydrogel optimization

**FIGURE 3:**
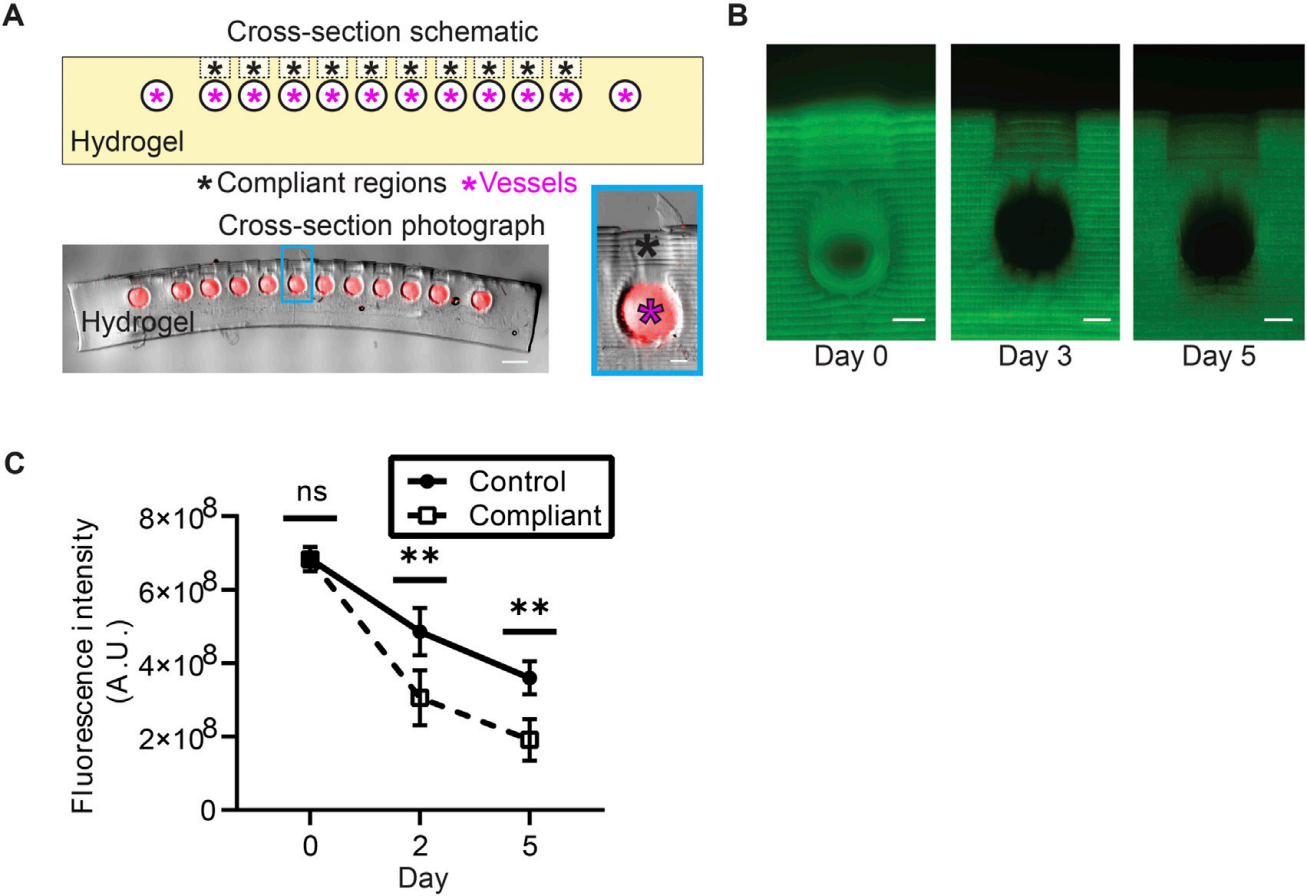
Hydrogel design featuring compliant regions above blood vessel segments. **(A)** Top: lengthwise cross-section schematic of the hydrogel showing intended compliant regions (black asterisks) above open vessels (magenta asterisks). Bottom: photograph of a thin section of a hydrogel (scale bar = 1 mm). Prior to sectioning and imaging, the artificial blood vessel was perfused with a crosslinkable solution containing 2 μm red fluorescent beads (see methods). Photograph is an overlay of a phase-contrast image and an image obtained through fluorescence microscopy. The highlighted blue region is expanded on the right to emphasize a patent channel and associated compliant region (scale bar = 200 μm). The vessel is less than 700 μm from the surface of the hydrogel to enable mosquito feeding. **(B)** Cross-sections of hydrogels with 150 kDa FITC-dextran incorporated in the pre-polymer solution. Imaging these hydrogels over several days reveals that the FITC-dextran elutes from hydrogels faster in the compliant regions than it does in neighboring unmodified (control) regions (scale bars = 200 μm). **(C)** Quantification of FITC-dextran elution from compliant and neighboring control regions of hydrogels over time. Significant differences between fluorescence intensities in compliant and control regions were observed after 2 days (*p* < 0.01) and 5 days (*p* < 0.01) of eluting in PBS (Mann-Whitney test).

Early attempts to induce mosquito feeding with these PEGDA-GelMA hydrogels revealed that mosquitoes struggled to puncture the hydrogel deeply enough to reach the channel. A mosquito proboscis is long enough to reach artificial vasculature, implying that vessel depth is not the issue. To address this problem, the surface of these hydrogels was selectively softened directly above the blood channel to increase their compliance (Figure 3A, see methods). Analysis of these compliant regions showed that they are more porous than adjacent regions of the hydrogel surface, as confirmed by a diffusion assay (Figure 3B, C, see methods). Although this change did not drastically change the overall number of feedings, mosquitoes appeared to have less difficulty consuming a blood meal. We therefore incorporated compliant regions into all subsequent hydrogels. The fact that mosquitoes were able to feed on our hydrogels confirmed the feasibility of our material as a mosquito food source.

**FIGURE 4:**
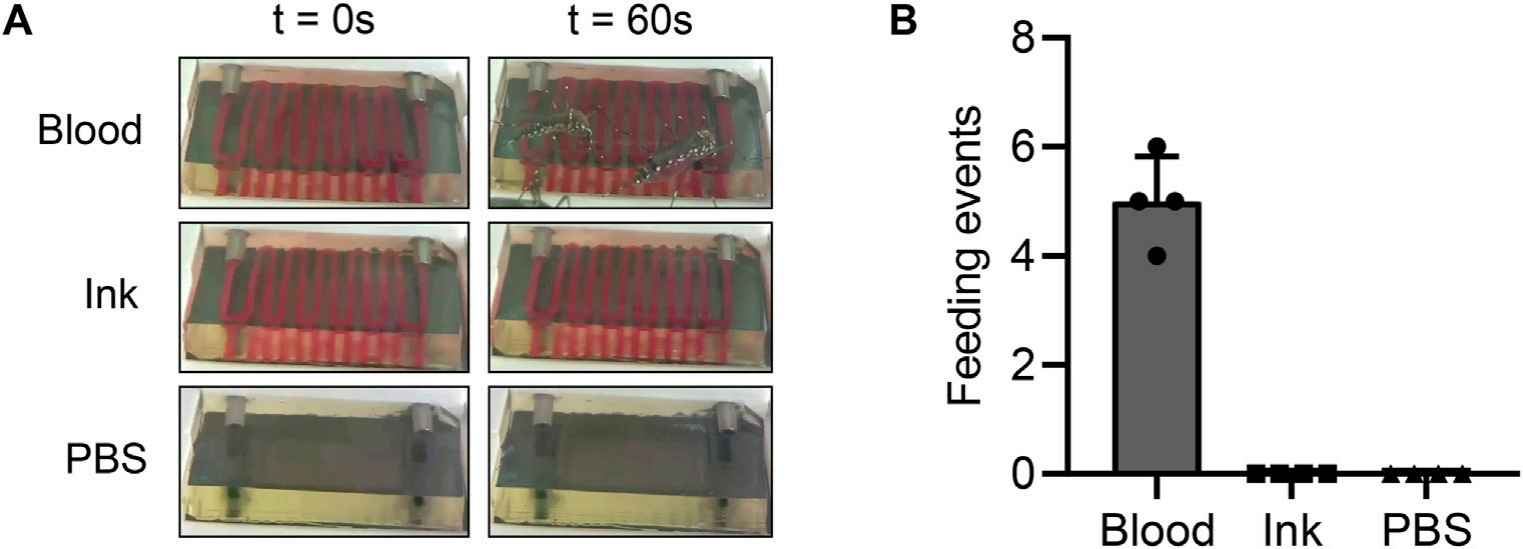
Perfused fluid composition impacts mosquito attraction **(A)** Images showing hydrogels perfused with blood, India ink, or PBS at the beginning of an experiment and 1 minute after introducing mosquitoes. The blood- and India ink-perfused hydrogels are essentially indistinguishable from each other, allowing us to examine whether the color contrast between blood and hydrogels attracts mosquitoes. **(B)** Across a total of 3 repeated experiments, mosquitoes only fed on the hydrogels containing blood. This result confirms that a chemical component of blood attracts mosquitoes.

Although we previously demonstrated that mosquitoes could feed on our hydrogels, it was unclear whether mosquitoes were attracted to the blood itself, the color contrast between the blood and the translucent hydrogels, or a property of the hydrogels themselves. To disentangle these variables, we tested the extent to which blood successfully attracted mosquitoes to our hydrogels. In three independent feeding cages, we offered *Ae. aegypti* mosquitoes hydrogels perfused with blood, red India ink, or PBS and observed their feeding tendencies. In this experiment, the PBS acted as a negative control—since it does not contain any sugars and is isotonic with the hydrogels, any mosquito attraction to these hydrogels is likely not due to the perfused liquid. This experiment showed that mosquitoes only fed on the hydrogels containing blood, as expected (Figure 4; Table 1). Although mosquitoes spent a substantial amount of time around the hydrogels in both of the other experimental groups, none of them engaged in host-seeking behavior. We therefore concluded that a chemical component of blood, rather than its visual appearance, attracts mosquitoes. This experiment also confirmed that hydrogels do not inherently attract mosquitoes, indicating that no further optimization of hydrogel composition is necessary.

**TABLE 1:** Effect of perfused fluid on mosquito attraction.

Perfused fluid	Engorged mosquitoes	Unfed mosquitoes	Chi-square test for equal proportions	
Blood	20	111	Chi-square	34.84
India ink	0	107	Df	2
PBS	0	108	*p*-value	<0.0001
Total	20	326		

###  Repellent screening

**FIGURE 5:**
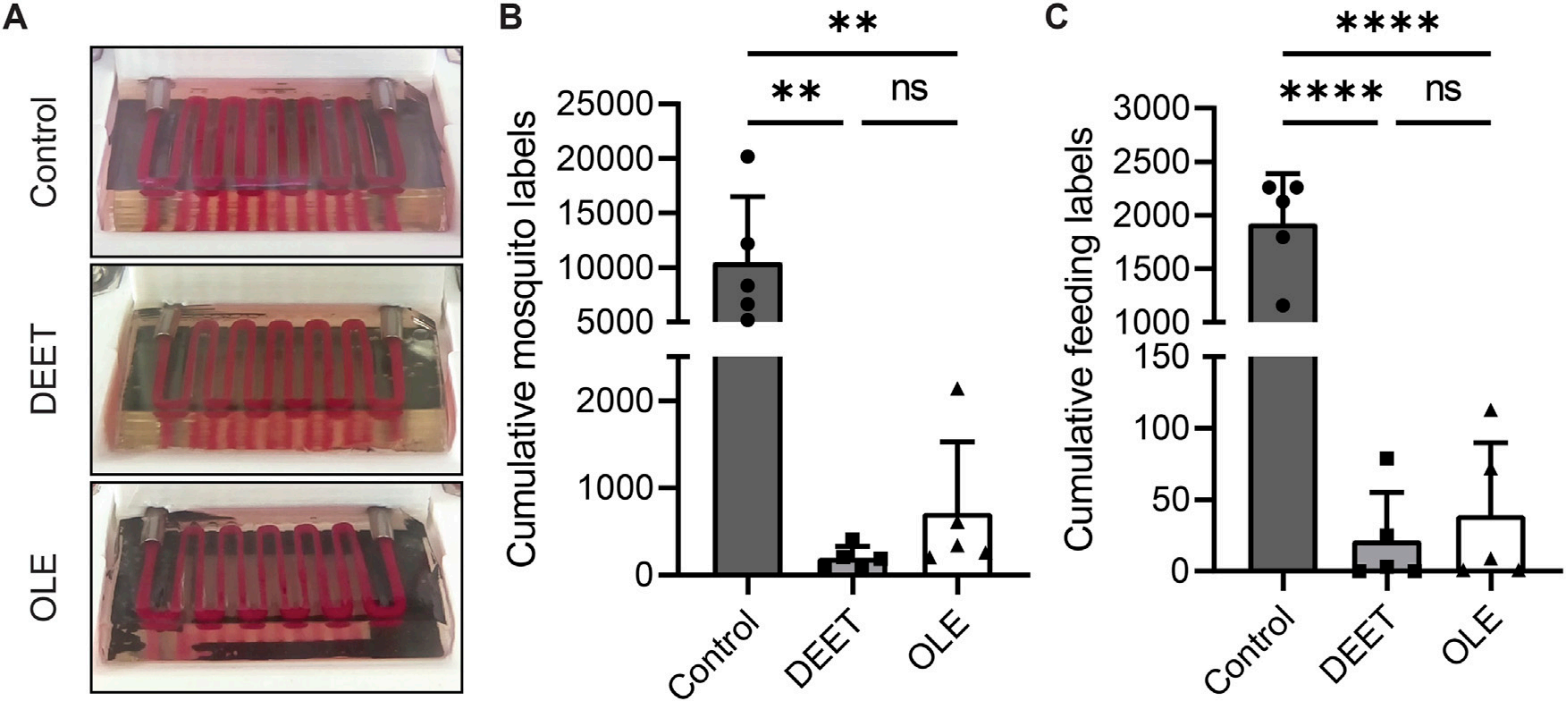
Evaluation and computer vision modeling of repellent screen. **(A)** Representative images of video data for the control, DEET, and lemon eucalyptus repellent groups. There is no apparent visual difference among the surfaces of the three groups. Photographs have been white balanced for visualization purposes. **(B)** The results of our machine learning model were reduced to a metric for each repellent screening replicate. The presence of mosquitoes near hydrogels dramatically declines when repellents are used, as expected. **(C)** The analysis in **(B)** was repeated for model detections of feeding/engorged mosquitoes. Repellents significantly decrease the number of feeding mosquitoes. The values reported in **(B–C)** reflect video downsampling by a factor of 16 (see methods).

To validate our feeding platform as a screening assay, we assessed the effectiveness of two available mosquito repellents as feeding deterrents. To ensure independence of experimental conditions, we increased the scale of our assay to include three isolated feeding cages which contained hydrogels coated with DEET, OLE, or PBS (Figure 5A, see methods). These screening experiments demonstrated that the DEET and OLE repellents significantly negatively impacted mosquito attraction (Table 2). There are no visible differences between hydrogels in each experimental group, indicating that DEET and OLE repel mosquitoes by non-visual mechanisms. In both experimental groups, none of the mosquitoes fed on any of the hydrogels. This experiment performed as expected, with mosquitoes avoiding hydrogels coated with repellent while feeding on control hydrogels.

**TABLE 2:** Effect of mosquito repellents on feeding behavior.

Repellent	Engorged mosquitoes	Unfed mosquitoes	Chi-square test for equal proportions	
Control	16	116	Chi-square	34.82
DEET	0	138	Df	2
OLE	0	138	*p*-value	<0.0001
Total	16	392		

We next investigated whether our machine learning model could expedite data analysis for our repellent screening experiments. Although previously explored visual representations of our model’s predictions can show striking differences between mosquito behavior in different videos (Supplementary Figure S2), further quantification would more convincingly demonstrate behavioral differences in unique experimental groups. To that end, we developed a metric to reduce our model predictions to a single numerical output for each label (see methods) using the frequency of machine learning model predictions as a proxy for mosquito activity and feeding activity. We then used this metric to analyze our repellent screening data. To filter out irrelevant data that manual tabulation would ignore anyway, we restricted our model’s detections to an area encompassing only hydrogels and their immediate surroundings (see methods). This approach yielded the same results and overall conclusions as with our manually tabulated data—both DEET and OLE effectively repelled mosquitoes and discouraged feeding on hydrogels (Figure 5C). Therefore, reducing our data to a single metric increases autonomy, decreases interpretation subjectivity, and improves throughput without altering experimental conclusions. The fact that this metric agrees with our manually tabulated observations (Table 2) and implies the same conclusions regarding the effectiveness of repellents suggests that our metric could be useful for other experiments in the future.

##  Discussion

In this study, we have developed a low-cost, scalable platform for collecting mosquito feeding data *via* recorded videos. This platform is compatible with blood from different sources, features 3D printed hydrogels that can be precisely modified, and can be used for different mosquito species. Each unit of our platform supports up to six hydrogels and theoretically enables testing of up to six experimental conditions at once, but we caution against simultaneously using different surface compounds (e.g. mosquito repellents) that alter conditions throughout an entire cage. There is also an opportunity to scale up our studies by using multiple copies of our existing cages in parallel, assuming there are enough cameras to adequately capture data. Hydrogel printing could be performed at a commercial scale at negligible cost, and hydrogels can be stored for months after synthesis in sterile and refrigerated conditions. Although we have not formally tested the upper and lower limits of how many mosquitoes can be reasonably introduced at once, our anecdotal evidence shows that introducing 20–30 female mosquitoes per cage yields the best results. We believe that part of the reason why we achieve relatively low feeding rates (13.8%) in our negative control group of repellent experiments is because our hydrogels can only accommodate a small number of mosquitoes on their surface at a time. Fortunately, the tunability and modularity of our platform allow us to experiment with increased hydrogel surface area in the future.

We have also created a machine learning model that detects the presence of mosquitoes, distinguishes between feeding/engorged and non-feeding mosquitoes, and can quantify mosquito presence and feeding over time. This model identifies mosquitoes within video frames at a rate that far exceeds what a human could achieve and does so more consistently because its identification relies on algorithms. Supplementation of model training data with augmented images (see Supplementary Methods), combined with natural laboratory variations in lighting and camera angles, makes the model more applicable to a variety of lab settings. An additional benefit of using a machine learning model is that its accuracy can be improved over time by adding more experimental data to it. Increasing the robustness of the model could also be achieved by including data from different experimental environments, which would expand the model’s applicability. Newly collected data from this platform can be easily transferred to a computer and subsequently to a cloud storage service, facilitating its incorporation into the machine learning model.

Previous studies have used a combination of object detection models, pose estimation, and object tracking to identify mosquitoes within video frames and identify feeding or engorged mosquitoes. Pose estimation has been used to demonstrate a remarkable ability to distinguish among different mosquito behavior patterns and quantify feeding over time (Hol et al., 2020). However, the experimental design in that study was only compatible with transparent meals, which drastically limits meal types and excludes blood entirely. As a result, it is unclear whether these results reflect mosquito feeding behaviors during blood ingestions. In a separate study, an object detection model was created to distinguish clustered from non-clustered mosquitoes when viewed from a steep camera angle (Wu et al., 2019). This model was then supplemented with pose estimation to track mosquito position. Both the object detection model and pose estimation results are impressive in terms of their accuracy, but this study did not attempt to evaluate feeding or identify feeding mosquitoes. Finally, object tracking software and a low-cost camera were recently used to evaluate the effectiveness of different repellents (Costa et al., 2022). The use of computer vision software to automatically quantify repellent effectiveness strongly aligns with our goals, but these authors do not distinguish between mosquito presence and feeding. Additionally, camera hardware size and resolution limits hindered this study’s scalability. We expand upon these studies by creating an object detection model that automatically identifies mosquitoes within videos and detects whether they have consumed a blood meal or not. To improve the translatability of this work, we use defibrinated blood as a food source instead of protein-deficient sources to closely mimic natural feeding conditions.

Our experiments successfully validated the repellent effects of DEET and OLE on mosquitoes and yielded extreme outcomes of feedings among our experimental groups. This study uses commercial concentrations of repellents, but we could investigate dose-dependent responses of established repellents and compare them to literature values (Xue et al., 2022). to validate our platform as a tool for screening different mosquito repellents in the future. Improving the resolution of our assay and demonstrating intermediate levels of mosquito attraction would enable identification of repellent candidates that are less potent than DEET or OLE for further investigation.

Although our platform is currently optimized for laboratory *Ae. aegypti* mosquitoes, it could be adapted for deployment in the wild for analysis of field mosquitoes. Wild mosquitoes often exhibit different feeding tendencies from laboratory mosquito strains, so studying wild mosquitoes is desirable because it more accurately represents pathogen-spreading mosquitoes. Deploying this platform in the wild would undoubtedly require overcoming several challenges such as a mobile power source and maintaining hydrogel moisture for long periods of time. We believe these challenges could be addressed with some effort, as several existing mosquito traps use car batteries as a power source. Furthermore, if an artificial protein source to replace blood were developed, it would obviate the need for blood in experiments and drastically reduce the risk associated with bloodborne pathogens. Precisely dispensing carbon dioxide near hydrogels could also substantially improve mosquito attraction in the wild, as some studies have demonstrated that carbon dioxide is critical for mosquitoes to locate hosts (Dekker and Cardé, 2011; McMeniman et al., 2014; Liu and Vosshall, 2019). Attraction could be further increased by coating hydrogels with chemicals associated with human skin (Bello and Cardé, 2022). Gathering data on field colonies of mosquitoes would yield results more representative of mosquitoes that spread the pathogens that cause diseases such as yellow fever and dengue. Adapting the platform to attract more mosquito species would further expand applicability to mosquitoes that carry pathogens like the malaria parasite. Based on our success with screening repellents, we presume that our platform could effectively compare different attractants as well.

In addition to overcoming hardware-related challenges associated with deploying our experimental setup in the wild, the computer vision model would require improvement as well. For this study, we have solely introduced one mosquito species at a time during experiments. While we feel confident that our model would be able to label mosquitoes from different species that are simultaneously present in a camera’s field of view, it was not designed to distinguish and independently label different mosquito species. Furthermore, mosquitoes were the only type of insect introduced to the machine learning model in these experiments. If our experimental setup were deployed in the wild, there is a high likelihood that other insects or arachnids may come into a camera’s field of view. In this scenario, we predict that our current model would either incorrectly identify these animals as mosquitoes or not label them at all. If other researchers want to apply our developed model to a wider range of animals or a less controlled environment, they will first need to train the model to identify species of interest. Additionally, it is possible that supplementing our model with pose estimation could elucidate other indicators such as body angle and proboscis penetration depth that would improve accuracy of feeding detections. Incorporating pose estimation would undoubtedly introduce more challenges, as the crowding mosquitoes used in some of our training data would obscure body parts vital for pose estimation. However, pose estimation would enable quantification of metrics that object detection struggles with, such as probing time or number of probing events before mosquitoes begin feeding. Supplementing an object detection model with pose estimation could also reduce the chances that mosquitoes are labeled as both feeding and non-feeding simultaneously, which is a limitation of our current model. Although we are satisfied with our current model’s performance, it is possible that supplementary or entirely different algorithms would yield better results for the same application. Our machine learning model, data collection pipeline, and platform design are all amenable to scale-up, which would make future iterations of our experimental platform suitable for high-throughput screening assays for mosquito repellents.

##  Conclusion

The ability to examine multiple variables for their effect on mosquito feeding in a controlled setting substantially improves the impact and consistency of mosquito behavioral experiments. The richness of data that comes from an object detection model of feeding mosquitoes far exceeds what can be understood by aggregate data alone, and a machine learning model is more consistent and faster than manual quantification. This system is agnostic to the species of mosquito used and is compatible with blood from various animal sources, which enables matching blood source with mosquitos’ host preferences. Our mosquito feeding platform provides a more consistent, controlled method of collecting and rapidly analyzing mosquito behavioral data and can be used to investigate solutions to global health challenges.

## Data Availability

Our machine learning model is freely accessible at the following link: https://universe.roboflow.com/miller-lab-public/ae.aegypti_ver11-i1viw/dataset/3. Hydrogel and platform design files are available on Zenodo (doi: https://doi.org/10.5281/zenodo.7542453).
