# Supplementary material for: Development of an automated biomaterial platform to study mosquito feeding behavior
Source: Front Bioeng Biotechnol. 2023 Feb 9;11:1103748. doi: 10.3389/fbioe.2023.1103748 (PMC9946970; doi:10.3389/fbioe.2023.1103748)
Supplement: Supplementary file 1 [file DataSheet1.pdf]

Supplementary Material

Development of an automated biomaterial platform to study mosquito feeding behavior

Kevin D. Janson,[1] Brendan H. Carter,[2] Samuel B. Jameson,[2] Jane E. de Verges,[2] Erika S. Dalliance,[2] Madison K. Royse,[1] Paul Kim,[1] Dawn M. Wesson,[2*] Omid Veiseh[1*]

[1]Department of Bioengineering, Rice University, Houston, TX, USA.
[2]Department of Tropical Medicine, Tulane University, New Orleans, LA, USA.

1 Supplementary Methods

1.1 Polymer and photoinitiator synthesis

3.4 kDa poly(ethylene glycol) diacrylate (PEGDA) was prepared according to an established protocol (Miller et al., 2010). Poly(ethylene glycol) was added to triethylamine and acryloyl chloride in anhydrous dichloromethane, and was allowed to react overnight. For batch sizes up to 350 g, yields generally ranged between 80-90% and percent acrylation was verified as 99% by ^{1}H NMR for the characteristic methylene protons adjacent to the acrylate group.

Lithium phenyl-2,4,6-trimethylbenzoylphosphinate (LAP) was prepared according to an established protocol (Fairbanks et al., 2009). Dimethyl phenylphosphinite was reacted with 2,3,6-trimethylbenzoyl chloride overnight at room temperature under argon. This reaction was followed by the addition of 4 molar excess lithium bromide in 2-butanone, where the mixture was then heated to 50°C to allow the formation of a solid precipitate. The mixture was then cooled to room temperature over 4 hours and filtered using excess 2-butanone and diethyl ether. For batches up to 30 g, yields of up to 90% were achieved.

Gelatin methacrylate (GelMA) was synthesized according to an established protocol (Grigoryan et al., 2019). Gelatin was dissolved in a carbonate-bicarbonate buffer, and methacrylic anhydride was added dropwise for 3 hours. Temperature was maintained at 50°C. The solution was precipitated in ethanol for ~3 hours. The precipitate was dried, dissolved in PBS, frozen at -80°C, and lyophilized. GelMA solution was stored at -20°C until use.

1.2 Preliminary experiments and camera optimization

Preliminary video data was captured on a Sony Alpha a7rIV digital camera outfitted with a 90mm f/2.8 macro lens (B&H Photo Video, New York, NY). Aperture and shutter speed were adjusted to acquire the best data, but videos were consistently gathered at 30fps at 4K resolution. Hydrogels used for early experiments contained a vascular pattern, but the density of this pattern was increased in later iterations.

Preliminary videos acquired from this digital camera were reduced from 3840x2160 (4K) resolution to 1920x1080 (1080p) resolution for further analysis to reduce computation time. The videos gathered this way were very high quality, but this method is likely not scalable because of the high cost of the camera as well as the low number of mosquitoes that could be recorded at one time. Subsequent efforts were focused on minimizing the cost of recording equipment while preserving video data quality.

To reduce the cost of camera equipment, we assessed whether Raspberry Pi computers could be an acceptable substitute for costly professional cameras. These small, lightweight computers can be outfitted with small camera modules capable of recording at up to 1080p resolution at 30 frames per second (fps). Because 1080p resolution was sufficient for analysis in preliminary experiments, this resolution was deemed acceptable for future data collection. Additionally, some third-party manufacturers sell macro lenses compatible with Raspberry Pi camera modules. These lenses could serve as an adequate replacement for the costly macro lens used in preliminary experiments and would preserve the ability to gather high quality images of small objects like mosquitoes.

Replacing a professional camera with a Raspberry Pi computer not only reduced the cost of the recording equipment by two orders of magnitude, it also drastically reduced the required footprint of the camera. This reduction in occupied volume enabled multiplexing the Raspberry Pi camera setup because multiple Raspberry Pi computers and cameras still occupied less space than the original camera and tripod combination. By redesigning the perfusion chambers that hold the hydrogels in place during experiments, we successfully fit 6 hydrogels in the space previously occupied by one. Each of these hydrogels had a designated Raspberry Pi camera, enabling video capture with similar camera angle and quality as in preliminary experiments with a professional camera.

1.3 Egg count procedure

Following each feeding experiment, carbon dioxide was introduced to the glass cage until all mosquitoes were anesthetized. The mosquitoes were then gently aspirated and transferred to a cold plate or Flypad (Genesee Scientific, San Diego, CA, USA) for processing. Engorged females were placed in individual cages with a vial containing water and damp seed germination paper for egg deposition, as well as a source of 10% sucrose. Females were contained this way for 5-6 days in the insectary to allow egg deposition, after which the seed germination paper was collected and dried for 24 hours. Egg counts were tabulated by manual counting under a stereomicroscope and recorded. To evaluate whether feeding on blood flowed through hydrogels negatively impacted mosquitoes' ability to produce eggs, we compared egg counts of mosquitoes that fed on our hydrogels with a control group fed using standard laboratory practices (Supplementary Figure 3).

1.4 Training data augmentation

To increase model performance with a limited amount of data, the training dataset was supplemented with transformed images. A total of five augmentation transforms were used, and each augmented image contained all five applied transforms. Each image in the training dataset produced three augmented images during the first several model iterations. The transforms applied to each image are as follows:

- **Saturation** – color saturation was adjusted between -40% and +40% (uniformly distributed).
- **Noise** – up to 6% of pixels had their color altered.

- **Cutout** – six black squares each occupying 6% of the total image size were artificially overlaid in random positions. This augmentation increases model performance in cases where objects may be obstructed.
- **Mosaic** – four images were cropped to contain at least one label and these cropped images were stitched together to create a new image. This augmentation improves performance when detecting small objects.
- **Bounding box blur** – pixels within ground truth bounding boxes were slightly blurred.

1.5 Machine learning model architecture

Models were trained on the Roboflow Train and Deploy platform (available at https://roboflow.com/). The backend infrastructure has many proprietary technologies and its exact specifications cannot be revealed, but training results could be recreated with a similar YOLO backbone architecture such as YOLOv5-S if custom training was required.

YOLOv5-S (Jocher et al., 2020): Unlike the official release of YOLOv4, YOLOv5 currently exists in active development. Therefore, all YOLOv5 related code, and models may be subject to modification or deletion without notice. YOLOv5-S has 7.5 million parameters, 140 layers, and operates at a lightweight 7MB (14MB for weights pre-trained on COCO). This architecture uses the Cross Stage Partial Network (CSP) (Wang et al., 2019) as the processing backbone and was trained on MSCOCO to extract rich/informative features from an input image. YOLOv5 also uses a PANet (Liu et al., 2018) for the model-neck to generate feature pyramids and the computational friendly LeakyReLU and Sigmoid activation function. The model uses SGD as a default learning rate, but these tests were performed with the ADAM adaptive learning rate enabled (Kingma & Ba, 2014).

1.6 Computer vision training and quantification

To quantify the effectiveness of machine learning models, every image used during training was manually labeled with ground-truth labels. These ground-truth labels were used to benchmark model performance. Labels were applied for three object types: mosquitoes, abdomens of feeding/fed mosquitoes, and abdomens of non-feeding mosquitoes. All labels were drawn as bounding boxes of minimal size that still encompassed the object of interest. For mosquito labels, a box was drawn that included mosquitoes' eyes, thorax, and abdomen. Extremities such as legs, wings, antennae, and proboscises were only included if they happened to be within the bounding box that encompassed the other essential mosquito parts. Labels for abdomens of both feeding/fed and non-feeding were drawn to encompass the full visible abdomen. If mosquitoes were fully or partially obscured, labels were extended over the obstructing object as a good faith estimate of where the object would be. Partially obscured abdomens were labeled, but fully obscured abdomens were not.

Object detection machine learning models were evaluated based on precision (also known as positive predictive value), mean average precision (mAP), and recall (also known as sensitivity) (Baratloo et al., 2015; Safari et al., 2015). The formulas for each metric are as follows:

$$precision = \frac{TP}{TP + FP}$$

$$recall = \frac{TP}{TP + FN}$$

$$mAP = \frac{1}{n}\sum_{i=1}^{n} AP_i$$

Where TP = true positives, FP = false positives, FN = false negatives, and AP = average precision. Average precision is calculated for each class according to the following equation:

$$AP = \int_0^1 p(x)dx$$

Where p(x) is the precision-recall curve. True positives, false positives, and false negatives were counted according to an intersection-over-union (IoU) threshold of 0.5.

1.7 Heating experiments

Heating experiments were performed by offering *An. quadrimaculatus* mosquitoes three hydrogels heated to 34°C and three hydrogels at room temperature within a single cage. Hydrogel positions receiving heat were changed for each replicate according to a random number generator. Experiments were performed in triplicate. Blood was heated to 37°C before entering the cage. Mosquito preference between heated and unheated hydrogels was analyzed using a two-tailed unpaired t-test ($p<0.05$).

1.8 Mechanical testing of hydrogels

Mechanical testing of hydrogels was performed with a TA.XT Express texture analyzer (Stable Microsystems, Godalming, UK). Hydrogel disks with a diameter of 3 mm were fabricated and subjected to compression. Compression tests were performed with a 5 mm cylindrical steel probe tip to collect stress vs. compression distance. Disc heights were individually measured with a caliper to convert the compression distance data to strain. Young's modulus was then calculated by measuring the slope of the stress-strain curve within the elastic region. It was found that compliant regions did not significantly affect the Young’s modulus of bulk hydrogels, so we report a single Young’s modulus of 57.9 $\pm$ 2.4 kPa for hydrogels with identical material composition.

1.9 Surface characterization of hydrogels

The surface roughness of hydrogels was nondestructively assessed using optical profilometry. Hydrogels were fabricated as described previously. To improve visual contrast, hydrogels were stained before imaging using Amido Black 10B (aka Napthol Blue Black) in 1X PBS for 5 minutes prior to imaging. Hydrogels were then briefly rinsed in 1X PBS to remove excess dye. Profilometry measurements were acquired using a Zegage optical profilometer (Zygo Corp., Middlefield, CT, USA) and a computer equipped with ZeMaps v1.14.38 software. All measurements were taken using a 10x objective. Profilometry was performed at the Micro/Nano Fabrication Facility in the School of

Science and Engineering at Tulane University, a class 1000 clean room space. Surface roughness (R_a) was measured in both compliant regions and adjacent unmodified hydrogel regions (R_a for compliant regions = 13.292 µm, R_a for non-compliant regions = 0.0267 µm).

2 Supplementary Figures

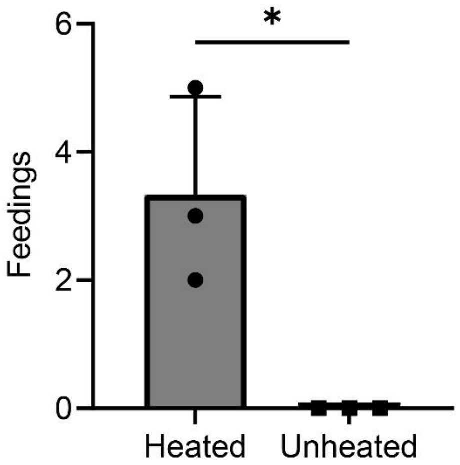

Supplementary Figure 1. Effects of PCB heating on *An. quadrimaculatus* mosquitoes. *An. quadrimaculatus* mosquitoes were offered six blood-perfused hydrogels, three of which were heated using our PCB. Mosquitoes exclusively fed on heated hydrogels, demonstrating that heat noticeably affects attraction for this species ($p<0.05$).

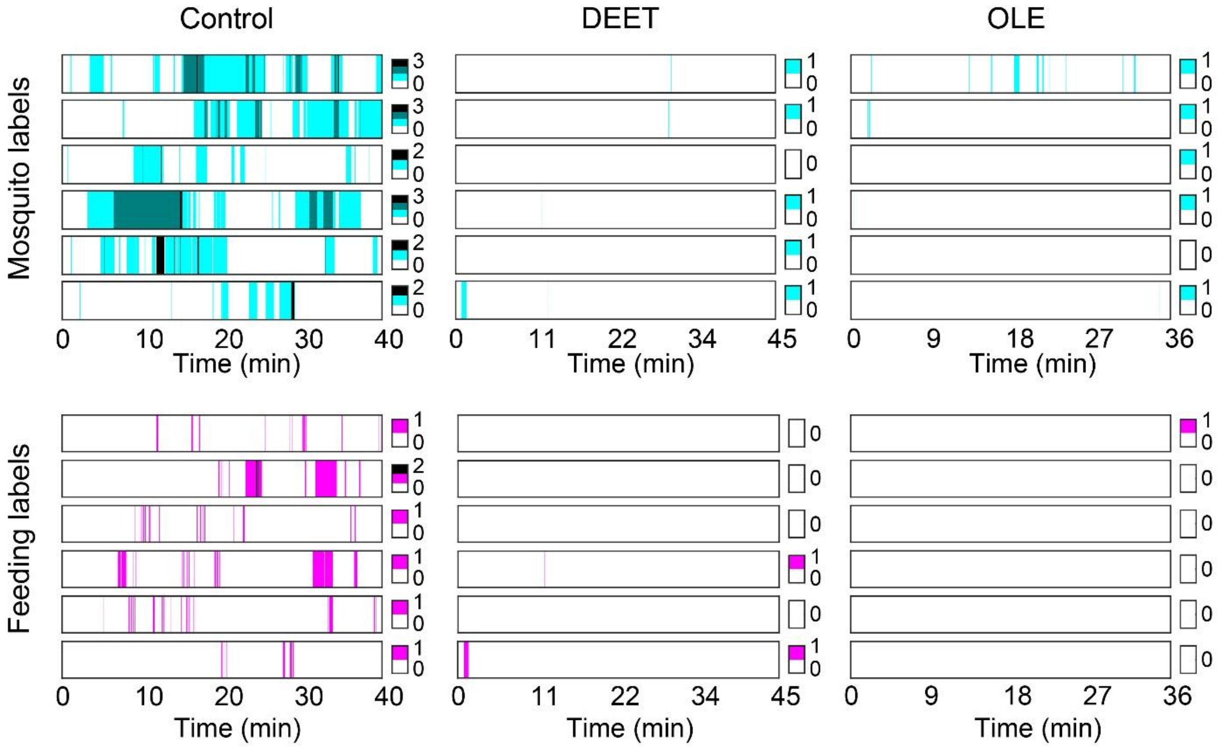

Supplementary Figure 2. Model predictions of mosquito presence and feeding activity in repellent screening experiments. The six plots in each section represent the six Raspberry Pi cameras used in each experiment. The videos used to generate these plots were restricted to a region of interest encompassing hydrogels and their immediate surroundings. The entire plot displays model predictions for the first replicate for this type of experiment. Both mosquito presence and feeding activity are noticeably higher in the control group than in either repellent group. This trend was consistently observed across all replicates in this study.

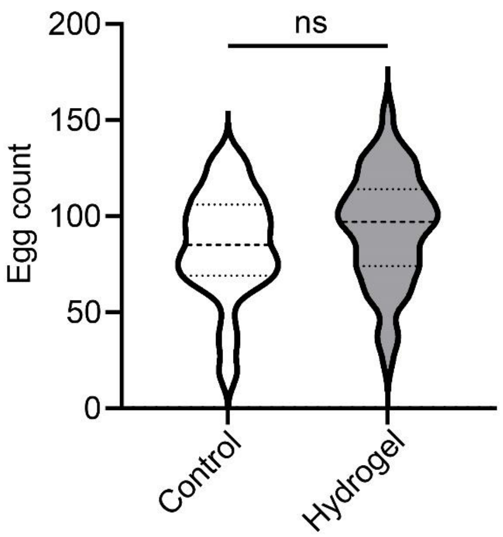

Supplementary Figure 3. Hydrogels do not substantially impact mosquitoes' ability to produce viable eggs. Violin plots compare egg counts of mosquitoes that fed on blood in a standard bell feeder (control, N=45) and those that fed on blood perfused through hydrogels (N=85) ($p>0.05$).

References

Baratloo, A., Hosseini, M., Negida, A., & Ashal, G. el. (2015). Part 1: Simple Definition and Calculation of Accuracy, Sensitivity and Specificity. *Emergency*, *3*(2), 48. /pmc/articles/PMC4614595/

Fairbanks, B. D., Schwartz, M. P., Bowman, C. N., & Anseth, K. S. (2009). Photoinitiated polymerization of PEG-diacrylate with lithium phenyl-2,4,6-trimethylbenzoylphosphinate: polymerization rate and cytocompatibility. *Biomaterials*, *30*(35), 6702–6707. https://doi.org/10.1016/j.biomaterials.2009.08.055

Grigoryan, B., Paulsen, S. J., Corbett, D. C., Sazer, D. W., Fortin, C. L., Zaita, A. J., Greenfield, P. T., Calafat, N. J., Gounley, J. P., Ta, A. H., Johansson, F., Randles, A., Rosenkrantz, J. E., Louis-Rosenberg, J. D., Galie, P. A., Stevens, K. R., & Miller, J. S. (2019). Multivascular networks and functional intravascular topologies within biocompatible hydrogels. *Science*, *364*(6439), 458–464. https://doi.org/10.1126/SCIENCE.AAV9750

Jocher, G., Stoken, A., Borovec, J., NanoCode012, ChristopherSTAN, Changyu, L., Laughing, Hogan, A., lorenzomammana, tkianai, yxNONG, AlexWang1900, Diaconu, L., Marc, wanghaoyang0106, ml5ah, Doug, Hatovix, Poznanski, J., yzchen. (2020). *ultralytics/yolov5: v3.0*. https://doi.org/10.5281/ZENODO.3983579

Kingma, D. P., & Ba, J. L. (2014). Adam: A Method for Stochastic Optimization. *3rd International Conference on Learning Representations, ICLR 2015 - Conference Track Proceedings*. https://doi.org/10.48550/arxiv.1412.6980

Liu, S., Qi, L., Qin, H., Shi, J., & Jia, J. (2018). Path Aggregation Network for Instance Segmentation. *Proceedings of the IEEE Computer Society Conference on Computer Vision and Pattern Recognition*, 8759–8768. https://doi.org/10.48550/arxiv.1803.01534

Miller, J. S., Shen, C. J., Legant, W. R., Baranski, J. D., Blakely, B. L., & Chen, C. S. (2010). Bioactive hydrogels made from step-growth derived PEG–peptide macromers. *Biomaterials*, *31*(13), 3736–3743. https://doi.org/10.1016/J.BIOMATERIALS.2010.01.058

Safari, S., Baratloo, A., Elfil, M., & Negida, A. (2015). Evidence Based Emergency Medicine Part 2: Positive and negative predictive values of diagnostic tests. *Emergency*, *3*(3), 87. /pmc/articles/PMC4608333/

Wang, C. Y., Mark Liao, H. Y., Wu, Y. H., Chen, P. Y., Hsieh, J. W., & Yeh, I. H. (2019). CSPNet: A New Backbone that can Enhance Learning Capability of CNN. *IEEE Computer Society Conference on Computer Vision and Pattern Recognition Workshops*, *2020-June*, 1571–1580. https://doi.org/10.48550/arxiv.1911.11929
